# Supplementary material for: Multidimensional Evaluation for Detecting Salt Tolerance of Bread Wheat Genotypes Under Actual Saline Field Growing Conditions
Source: Plants (Basel). 2020 Oct 6;9(10):1324. doi: 10.3390/plants9101324 (PMC7601346; doi:10.3390/plants9101324)
Supplement: Supplementary file 1 [file plants-09-01324-s001.pdf]

Table S1. Monthly average minimum temperature (Tmin, °C), maximum temperature (Tmax, °C), growing degree days (GDD, °C) and precipitation (Prec., mm) in the two growing seasons and 38 years' monthly averages (1983–2020).

Month	2018-2019				2019-2020				38 years average		
	Tmin	Tmax	GDD*	Prec	Tmin	Tmax	GDD	Prec	Tmin	Tmax	Prec
November	14.7	25.0	582	6.4	15.1	26.1	418	4.2	13.4	23.9	7.4
December	9.6	18.5	326	8.5	9.9	19.1	439	8.4	9.4	19.0	8.1
January	7.1	17.4	468	1.7	7.0	16.8	347	2.8	7.6	17.4	7.7
February	8.2	18.9	499	8.7	8.2	18.6	399	9.3	8.0	18.9	6.7
March	9.7	21.8	559	7.2	10.2	22.4	476	6.9	9.9	22.4	7.2
April	12.5	25.4	664	2.1	13.1	26.1	594	3.7	13.1	27.0	3.7

* Average monthly growing degree days was calculated based on a 0 ◦C baseline

Table S2. Chemical properties of soil and irrigation water of three wells at the experimental site. Data averaged over the two growing seasons.

Characteristics	Soil	Well 1	Well 2	Well 3
**Soil particles distribution**				
Sand (%)	86.95			
Silt (%)	8.75			
Clay (%)	4.30			
Textural class	Sandy loam			
Calcium carbonate ($CaCO_3$%)	57.99			
pH	8.15	7.82	7.96	7.66
Electrical conductivity (dS m^{-1})	7.74	5.25	8.35	11.12
**Saturation soluble extract cations and anions (mg 100g^{-1})**				
Calcium (Ca^{2+})	25.2	10.8	23.5	19.3
Magnesium (Mg^{2+})	5.7	7.2	14.5	18.8
Sodium (Na^{+})	57.8	53.6	66.1	105.1
Carbonate (CO_3^{-})	0.0	0.0	0.0	0.0
Bicarbonate (HCO_3^{-})	6.2	5.3	6.50	7.5
Chloride (Cl^{-})	61.9	39.1	57.66	93.1
Sulphate (SO_4^{-})	26.4	26.8	29.23	38.7

Table S3. Code, origin, and pedigree of eighteen used wheat genotypes.

Genotype	Code	Pedigree	Year of release	Origin
Giza-168	G1	MIL/BUC//SeriCM93046-8M-0Y-0M-2Y-0B	1999	Egypt
Gemiza-7	G2	CMH74 A. 630/5x//Seri 82/3/Agent CGM 4611-2GM-3GM-1GM-0GM	1999	Egypt
Gemiza-9	G3	Ald"S"/Huac"S"//CMH74A.630/5Xcgm4583-5GM-1GM-0GM	1999	Egypt
Gemiza-10	G4	MAYA74"S"/0N/1160-147/3/BB/GLL/4/CHAT"S"/5/CROW"S" CG5820-3G-1G-2G-0G	2004	Egypt
Sakha-94	G5	Opata/Rayon//KauzCMBW90Y3180-0T0PM-3Y-010M-010Y-10M-015Y-0AP-0S	2004	Egypt
Sids-12	G6	BUC//7C/ALD/5/MAYA74/ON//1160-147/3/BB GLL/4/HAT"S"/6/MAYA/VUL//CMH 74A.630/4*SX.SD7096-4SD- ISD- ISD-OSD	2008	Egypt
Gemiza-11	G7	Bow"s"/Kvz"s"//7c/seri82/3/Giza168/Sakha61CGM7892-2GM-1GM-2GM-1GM-0GM	2010	Egypt
Misr-1	G8	OASIS/KAUZ//4*BCN/3/2*PASTOP CMss00Y01881T-050M-030Y-030M-030WGY-33M-0Y-0S.	2010	CIMMYT
Misr-2	G9	SKAUZ/BAV92. CMss96M03611S-1M-0105Y-010M-010SY-8M—0y0S	2011	CIMMYT
Gemiza-12	G10	OTUS/3/SARA/THB//VEE CCMSS97Y00227S-5Y-010M-010Y-010M-2Y-1M-0Y-0GM	2013	Egypt
Shandawel-1	G11	SITE//MO/4/NAC/TH.AC//3*PVN/3/MIRLO/BUC. CMss93B00567S-72Y-010M-010Y-010M-3Y-0M-0THY-0SH.	2013	CIMMYT
Giza-171	G12	SAKHA 93 / GEMMEIZA 9 S.6-1GZ-4GZ-1GZ-2GZ-0S	2014	Egypt
Sids-14	G13	SW8488*2/ KUKUNACGSS01Y00081T-099M-099Y-099M-099B-9Y-0B-0SD	2018	Egypt
Line-6052	G14	KIRITATI/ WBLL1. CGSS02Y00138S-099M-099Y-099M-44Y-0B	-	CIMMYT
Line-6078	G15	WAXWING*2/4/SNI/TRAP#1/3/KAUZ*2TRAP//KAUZ. CGSS01B00055T-099Y-099M-099M-099Y-099M-64Y-0B	-	CIMMYT
Line-6083	G16	WAXWING*2/KUKUNA. CGSS01B00057T-099Y-099M-099M-099Y-099M-11Y-0B	-	CIMMYT
Line-6084	G17	WAXWING*2/KUKUNA. CGSS01B00057T-099Y-099M-099M-099Y-099M-13Y-0B	-	CIMMYT
Line-1208	G18	ICB97-0727-0AP	-	CIMMYT

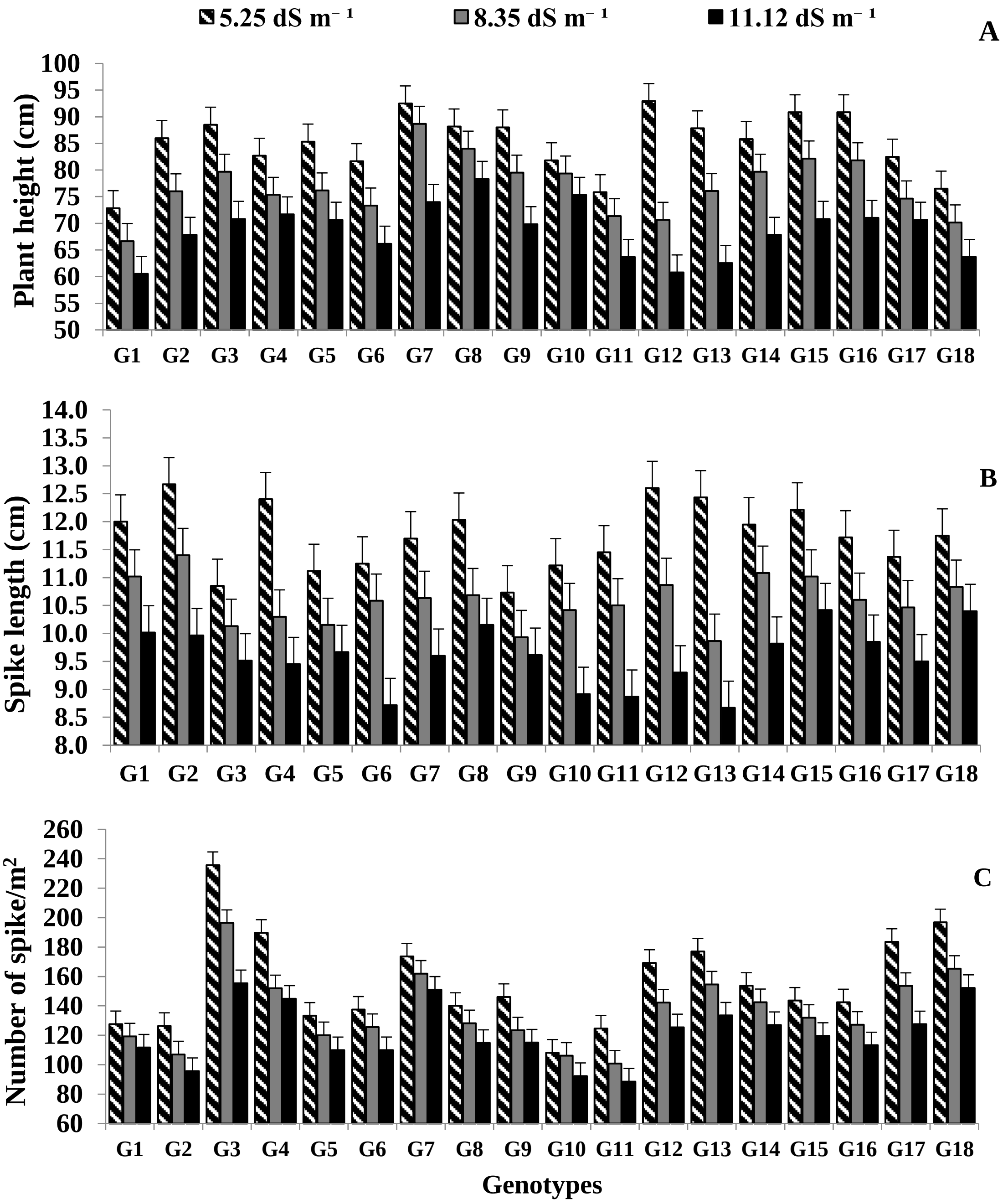

Figure S1. Impact of different salinity levels on plant height (**A**), spike length (**B**), and number of spikes m^{-2} (**C**) for 18 wheat genotypes. The bars on the top of the columns represent the LSD ($p <$ 0·05). Codes of the genotypes are illustrated in Table S3.

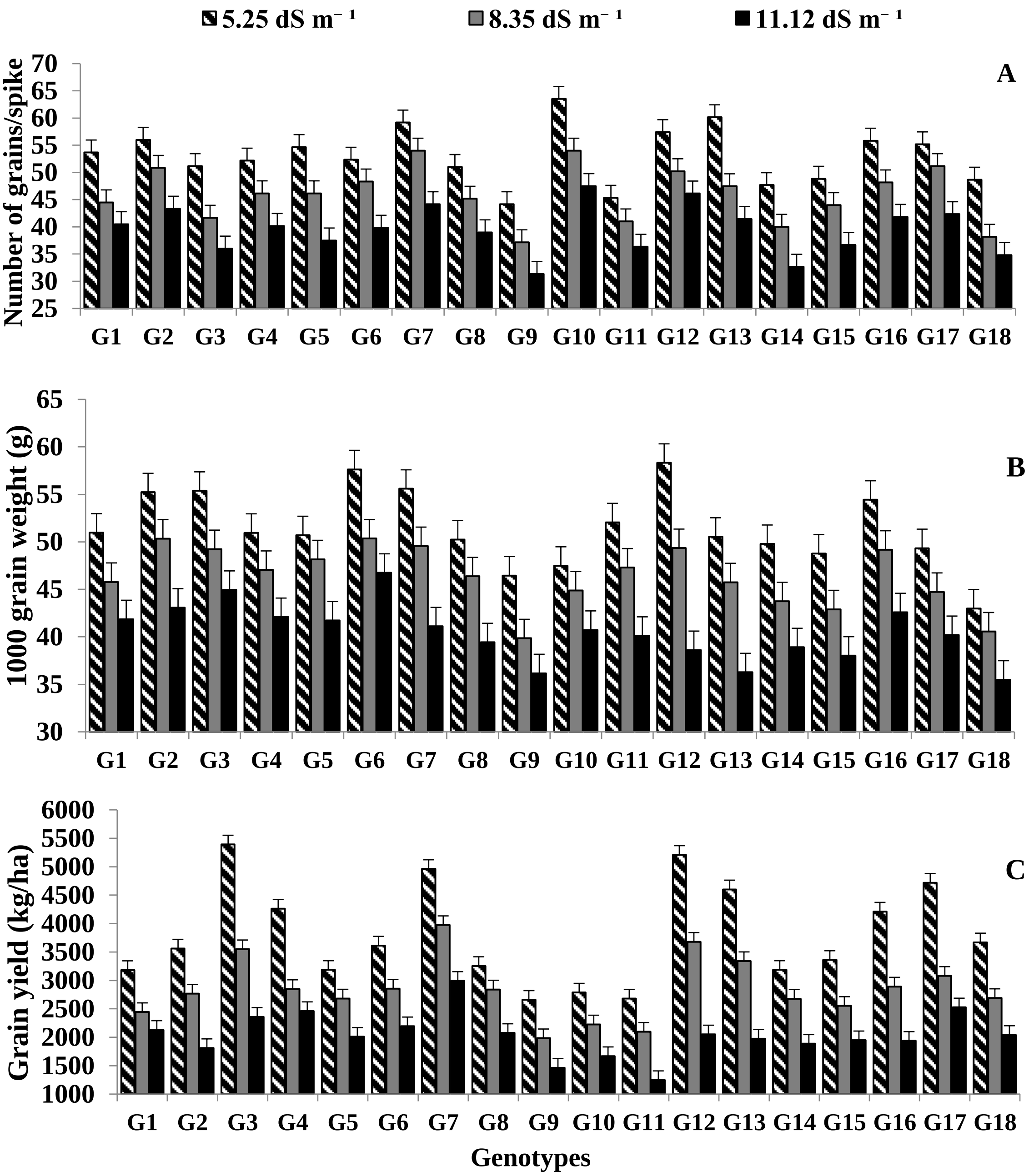

Figure S2. Impact of different salinity levels on the number of grains per spike (**A**), 1000-grain weight (**B**) and grain yield ha^{-1} (**C**) for 18 wheat genotypes. The bars on the top of the columns represent the LSD ($p < 0{\cdot}05$). Codes of the genotypes are illustrated in Table S3.
